# Potential of Natural Phenolic Compounds as Antimicrobial Agents against Multidrug-Resistant *Staphylococcus aureus* in Chicken Meat

**DOI:** 10.3390/molecules28186742

**Published:** 2023-09-21

**Authors:** Alaa Eldin M. A. Morshdy, Karima M. E. Abdallah, Heba E. Abdallah, Fahad D. Algahtani, Mohamed Tharwat Elabbasy, Suleman Atique, Khursheed Ahmad, Mohammad A. A. Al-Najjar, Hossam M. Abdallah, Abdallah Fikry A. Mahmoud

**Affiliations:** 1 Food Hygiene, Safety, and Technology Department, Faculty of Veterinary Medicine, Zagazig University, Zagazig 44519, Egypt; ammorshdy@vet.zu.edu.eg (A.E.M.A.M.); hebarashed14710@gmail.com (H.E.A.); 2 Department of Public Health, College of Public Health and Health Informatics, University of Hail, Ha’il 81451, Saudi Arabia; 3 College of Medicine, University of Ha’il, Ha’il 55473, Saudi Arabia; 4 Department of Public Health Science, Faculty of Landscape and Society, Norwegian University of Life Sciences, 1430 Ås, Norway; 5 Department of Biotechnology, Yeungnam University, Gyeongsan 38541, Republic of Korea; 6 Faculty of Pharmacy, Applied Science Private University, Amman 11193, Jordan; moh_alnajjar@asu.edu.jo; 7 Department of Microbiology, Faculty of Veterinary Medicine, Zagazig University, Zagazig 44519, Egypt; hmmuhamed@vet.zu.edu.eg

**Keywords:** antimicrobials, chicken meat, foodborne pathogen, multidrug resistance, phenolic compounds, *Staphylococcus aureus*

## Abstract

**Simple Summary:**

*Staphylococcus aureus* is a prevalent foodborne bacterium causing significant morbidity, mortality, and economic loss, due especially to pathogenic and multidrug-resistant (MDR) strains. This study focused on determining the prevalence of *S. aureus* in chicken meat in Egyptian markets and assessing the effectiveness of natural phenolic compounds as novel antibacterial agents against MDR *S. aureus*. The study found that 91.3% of chicken meat parts were contaminated with *S. aureus*, with 81.8% of the isolates being MDR. Twenty-two antibiotic resistance patterns were identified, and six strains showed the highest combination of virulence and resistance genes. The phenolic compound hydroquinone exhibited the most potent antibacterial activity compared to other compounds, making it a potential alternative to conventional antibiotics against the pathogenic MDR *S. aureus* found in raw chicken meat. This study highlights the need for urgent interventions to improve hygiene for safer meat in Egyptian markets and suggests hydroquinone as an effective natural compound for inhibiting foodborne pathogens.

**Abstract:**

*Staphylococcus aureus* is one of the most widespread foodborne bacteria that cause high morbidity, mortality, and economic loss, primarily if foodborne diseases are caused by pathogenic and multidrug-resistant (MDR) strains. This study aimed to determine the prevalence of *S. aureus* in chicken meat in Egyptian markets. Thus, this study might be the first to assess the efficiency of different natural phenolic compounds as novel antibacterial agents against MDR *S. aureus* pathogens isolated from raw chicken meat in the Egyptian market. The incidence and quantification of pathogenic *S. aureus* were detected in retail raw chicken meat parts (breast, thigh, fillet, and giblets). In total, 73 out of 80 (91.3%) of the chicken meat parts were contaminated, with *S. aureus* as the only species isolated. Of the 192 identified *S. aureus* isolates, 143 were coagulase-positive *S. aureus* and 117 isolates were MDR (81.8%, 117/143). Twenty-two antibiotic resistance profile patterns were detected. One strain was randomly selected from each pattern to further analyze virulence and resistance genes. Extracted DNA was assessed for the presence of antibiotic-resistance genes, i.e., vancomycin-resistance (*vanA*), aminoglycosides-resistance (*aacA–aphD*), apramycin-resistance (*apmA*), and methicillin-resistance (*mecA*), penicillin-resistance (*blaZ*), and virulence genes staphylococcal enterotoxins (*sea* and *seb*), Panton–Valentine leucocidin (*pvl*), clumping factor A (*clfA*), and toxic shock syndrome toxin (*tst*). Clustering analyses revealed that six *S. aureus* strains harbored the most virulence and resistance genes. The activity of hydroquinone was significantly higher than thymol, carvacrol, eugenol, and protocatechuic acid. Therefore, phenolic compounds, particularly hydroquinone, could potentially alternate with conventional antibiotics against the pathogenic MDR *S. aureus* inhabiting raw chicken meat. Hence, this study indicates that urgent interventions are necessary to improve hygiene for safer meat in Egyptian markets. Moreover, hydroquinone could be a natural phenolic compound for inhibiting foodborne pathogens.

## 1. Introduction

Foodborne diseases (FBDs) arise from the consumption of food containing physical, chemical, or biological hazards, with most infections caused by biological agents such as bacteria, viruses, and parasites. FBDs pose significant health risks and present obstacles to sustainable development in all countries, particularly in developed countries [1,2]. The most vulnerable groups to FBDs include the elderly, those with chronic illnesses, and kids. In developed countries with a high population density and inadequate hygienic standards for handling fresh meat, the risk of FBD is especially pronounced [3]. The economic burden of hazardous food in developed countries amounts to an annual loss of over USD 110 billion, encompassing productivity losses and medical expenses [4]. Animal-source foods (ASF) play a vital role in providing essential nutrients in a palatable and easily digestible form. However, they can also serve as a vector for the transmission of common infections and toxins produced by various pathogens [5,6].

Among the main causes of foodborne illnesses, pathogenic bacteria, especially Gram-positive *Staphylococcus aureus*, are prevalent in ASF. *S. aureus*, typically found as a commensal bacterium in humans, can act as an opportunistic pathogen, leading to a wide range of diseases from minor skin infections to severe and even fatal conditions [7]. In particular, human pathogens *S. aureus* and several coagulase-positive staphylococci (CPS) species, including foodborne diseases, produce various enterotoxins that can result in diverse clinical symptoms [8]. Despite proper cooking, most staphylococcal enterotoxins (SEs) are heat-resistant and can still cause illnesses when ingested [9]. Chicken meat is a common source of *S. aureus* contamination, necessitating monitoring and control of its incidence in retail raw chicken meat, especially in developed countries [10].

*S. aureus* exhibits a notable diversity in virulence and resistance genes, contributing to its rapid and aggressive pathogenicity [11]. The emergence of multidrug resistance (MDR) has posed significant challenges to effective therapies and has escalated in recent times [12,13]. The prevalence of MDR varies depending on the microbial species and its source [14,15]. Notably, MDR has become a pressing concern in animal-origin foods, particularly in chicken meat products. The transmission of MDR *S. aureus* in the food industry has led to far-reaching economic, social, and health implications [16]. Zoonotic *S. aureus* strains have been found to harbor antibiotic-resistance genes, reducing the effectiveness of commonly used antibiotics in veterinary treatment [17,18]. Abolghait et al. [19] reported that *S. aureus* exhibits a wide array of virulence genes. Moreover, the contamination of raw chicken meat and poultry products with *S. aureus* and its enterotoxins can pose a health risk, especially when stored at room temperature. Therefore, it is crucial to investigate the prevalence of resistance and virulence genes in the pathogens present in raw chicken meat to gather data on the extent and progression of this problem and to explore effective management strategies.

In addition, bacterial biofilm development is another critical element contributing to a severe problem during infection treatment [20]. Biofilms are populations of mono- or multi-bacterial species encased in a protective extracellular matrix [21]. Exopolysaccharides, lipids, surfactants, environmental DNA, proteins, and water are standard biofilm matrix components [22]. Bacterial colonies may adhere and remain on inanimate surfaces and within the body because of the forming biofilm. The first stage in biofilm development is the adhesion of bacteria to various surfaces with the assistance of exopolysaccharides, surface proteins, fimbriae, and pili [23]. Following the formation of the mature biofilm, the bacteria within this protective structure will exhibit various metabolic states. Specifically, the bacteria in the surface layer of the biofilm will be aerobic and metabolically active. In contrast, the bacteria in the deeper layers are fermentative and dormant due to nutrient deficiency and lower oxygen concentrations [24]. A thick biofilm also serves as a pharmacokinetic barrier, limiting the diffusion of antimicrobial agents and other toxic substances [25]. In essence, biofilms offer double protection against antibiotics because most antibiotics are only effective against actively replicating (i.e., planktonic) cells. Therefore, bacteria entrenched in biofilms may have minimum inhibitory concentrations (MICs) that are 10–10,000 times higher [24]. Unsurprisingly, biofilms are an essential virulence factor in developing bacterial pathogenicity, given their resistance to antibiotics and protective qualities against harsh environmental stressors (such as sheer forces drying) and the immune system (such as phagocytosis). Hence, combating these persisters is a real challenge and calls for innovative antimicrobial agents.

Consumers and the food industries are paying much attention to the use of natural antibacterial agents in the food industry. This is due to three essential aspects. First, the group of microorganisms, including foodborne pathogens, that are not only antibiotic-resistant but also more tolerant to various food processing and preservation methods has dramatically increased due to the abuse and improper management of antibiotics. Researchers are now more interested in creating and using natural ingredients in meals due to consumers’ growing knowledge of the possible health risks associated with synthetic preservatives compared to the advantages of natural additives. The second aspect represented is that this has compelled the food industry to search for substitute preservatives to improve food quality and safety. Because natural phenolic compounds exhibit antibacterial qualities that are effective against a wide variety of foodborne pathogens, substances produced from natural sources have the potential to be utilized to ensure the safety of food. The third factor is their bioactivities, such as antioxidant, anti-aging, cardioprotective, anticancer, and anti-inflammatory properties [26].

Phenolic compounds, naturally present in essential oils, serve as common antibacterial agents [27]. Depending on their structure and concentration, these compounds can either promote or inhibit the growth of microorganisms [28,29,30]. Notably, phenolic compounds are not only known for their antibacterial properties but also their antioxidant capabilities as free radical scavengers. The extent of their antioxidant capacity is determined by the number and arrangement of their hydroxyl groups and the level of structural conjugation [31]. Furthermore, phenolic compounds interact with various chemicals involved in microbial/bacterial metabolism [32]. They inhibit bacterial growth at several levels and through various metabolic pathways, affecting the structure and synthesis of nucleic acids, the composition of the cell membrane, and the activity of numerous enzymes [33,34]. Given these diverse bioactivities, including flavoring and antioxidant and antimicrobial effects, the food industry has become interested in using phenolic compounds as reagents to potentially reduce the overall quantity of food additives [35,36].

In Egypt, most citizens regularly buy fresh food, particularly fresh chicken meat, from traditional markets commonly known as wet markets [37]. These traditional markets in Egypt resemble those in neighboring countries like Libya, Tunisia, Sudan, Morocco, and Jordan, where food safety standards for animal-source foods (ASF) are not yet up to par. Previous studies have highlighted the poor hygiene practices among meat sellers and slaughterhouses in Egypt, where handling and slaughtering techniques are often based on age-old customs that may not prioritize hygiene [38,39]. For example, slaughtering processes are frequently carried out in inadequately controlled and unhygienic environments. Additionally, the unsanitary handling and transportation of meat and basic slaughterhouse facilities may facilitate meat contamination with pathogenic bacteria as it moves through the food chain to formal and informal retail markets. Various factors contribute to bacterial contamination and proliferation in retail chicken meat, including inadequate infrastructure, improper washing and disinfection procedures, haphazard handling of contaminated materials, and a lack of temperature control [40].

In contrast, supermarkets typically maintain better standards, with access to clean water, refrigeration systems, and suitable processing facilities. As a result, poultry products sold in supermarkets may be comparatively safer than those in traditional marketplaces. However, supermarkets are not as prevalent in Egypt as in some high-income countries. Moreover, data on foodborne disease burdens in developing countries like Egypt, Cambodia, and Libya are scarce, as monitoring efforts are relatively limited compared to high-income nations with more extensive information resources [1].

Limited research has explored the antimicrobial activity of phenolic compounds against zoonotic MDR *S. aureus* strains with different virulence characteristics. Most existing research on phenolics’ effects has primarily focused on just one or two reference strains [41]. To this end, this study aims to identify the prevalence of MDR and virulent *S. aureus* strains in chicken meat from Egyptian markets, while also assessing the presence of resistance and virulence genes within these strains. Additionally, the study aims to evaluate the bioactivity of natural phenolic compounds as antimicrobial and antibiofilm agents. The findings of this research will provide valuable insights for food safety management in the Egyptian market, shedding light on the occurrence of pathogenic MDR *S. aureus* strains. Moreover, this study proposes a viable approach to combat foodborne *S. aureus* pathogens by utilizing natural phenolic compounds as alternative antibacterial agents instead of conventional antimicrobial agents.

## 2. Results

### 2.1. Phenotypic Characterizations and Incidence of CPS

**Figure 1:**
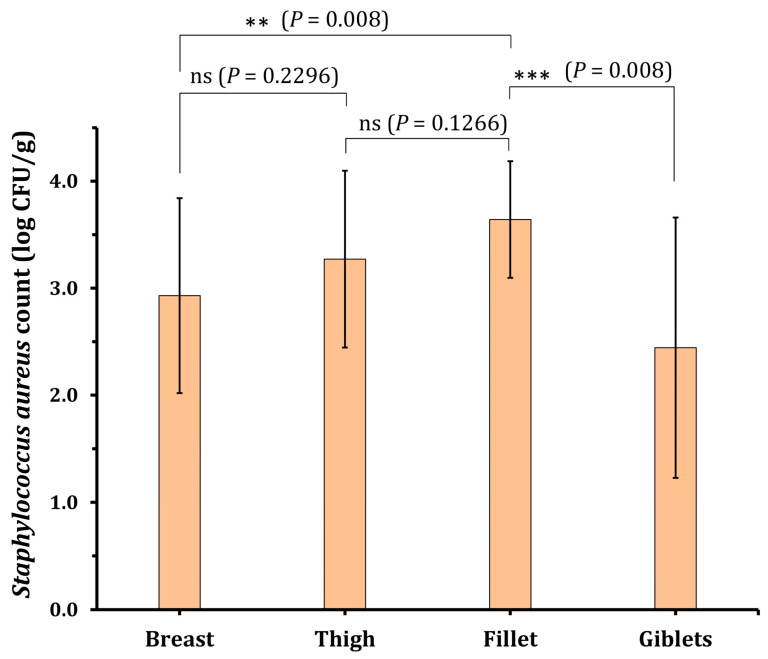
The incidence of *S. aureus* in different chicken meat samples and the statistical analysis using a *t*-test showing the significant variation in the contamination between the tested retail raw chicken meat samples.

Among the 80 cut-up chicken meat samples tested, 73 (91.3%) were found to be contaminated by *S. aureus* as the only isolated species. The incidence of CPS was detected in 100% (20/20) of the breast, 95% (19/20) of the thigh, and 85% (17/20) of both the giblet and fillet samples (Table 1). Out of the 192 *S. aureus* isolates screened from the investigated chicken meat samples, 143 isolates were identified phenotypically as CPS according to their growth on BP-EY agar and hemolysis and coagulase activities as confirmed by the MALDI TOF MS Biotyper system; 44 (30.8%) were from giblets, 38 (26.6%) from breasts, 33 (23.1%) from thighs, and 28 (19.6%) from fillets (Figure 1). The statistical analysis revealed no significant difference in the mean of total staphylococcal count between the examined samples of fillet and thigh (*p* = 0.1266). Also, there was an insignificant variation between the CPS count in the thigh and breast samples (*p* = 0.2296). However, there was a significant variation between the CPS count in the fillet and giblet samples at *p* = 0.0008 and between the fillet and breast samples at *p* = 0.008.

### 2.2. Coagulase-Positive Staphylococci Incidence

As illustrated in Table 1, the obtained data showed that the staphylococcal count in the analyzed samples ranged from 0.9 to 4.8 with an average value of 2.9 ± 0.9 log CFU/g for chicken breast and ranged from 1.55 to 4.8 with an average value of 3.3 ± 0.8 log CFU/g for chicken thigh samples. Also, the CPS count for chicken giblet samples ranged from 0.48 to 4.12 with an average value of 2.4 ± 1.2 log CFU/g and from 2.8 to 4.4 with an average value of 3.6 ± 0.5 log CFU/g for chicken fillet (Table 1). Table 1 also showed that the average CPS count for the investigated samples ranged from 0.48 to 4.8, with an average value of 3.1 ± 0.9 log CFU/g. According to the Egyptian Organization for Specification and Quality Control [42] and the Health Protection Agency [43], these findings reflect that only 13.7% (10/73) of the investigated samples were satisfactory and 63% (46/73) were unsatisfactory. Moreover, 23.3% (17/73) were unacceptable.

### 2.3. Antimicrobial Susceptibilities Test

The AST of 143 *S. aureus* strains against 11 antimicrobial agents was investigated. As shown in Table 2, the occurrence of resistance of *S. aureus* strains against CTX was considerably higher in comparison with other antimicrobial agents. The maximum resistance was observed against CTX (70.6%), followed by IMP (66.4%) and PMB (65.7%), GEN (60.1%), ERY (58.7%), SAM and CAF (51%), and CHL (50.3%). The resistance to FA was 42.7%. The resistance to VAN and TET was 34.3% and 31.5%, respectively, as shown in the Supplementary Data (Figure S1). Antibiogram resistance profiles (ARS) of isolated *S. aureus* strains showed that out of 143 *S. aureus* strains, 117 were MDR (strains showed resistance to three or more antimicrobial classes), while 5 (CPSA-110, CPSA-39, CPSA-47, CPSA-67, and CPSA-94) were classified as pan drug resistant strains (resistant to all antimicrobial classes). The MAR index analysis revealed that all tested strains had a very high MAR index value of more than 0.2 (Table 3).

### 2.4. Molecular Characterization of S. aureus

**Figure 2:**
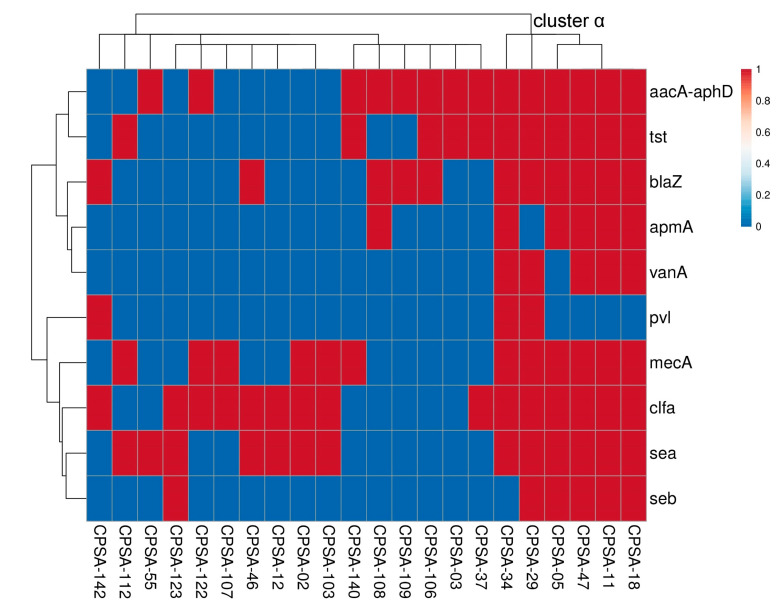
Clustering analysis showing the incidence of resistance and virulence genes in tested MDR *S. aureus* strains isolated from different chicken meat samples. *mecA*–methicillin-resistance, *blaZ*–penicillin-resistance, *vanA*–vancomycin-resistance, *apmA*–apramycin-resistance, *aacA*–*aphD*–aminoglycosides-resistance, *sea* and *seb*–virulence genes staphylococcal enterotoxins, *pvl*–Panton–Valentine leucocidin, *clfA*–clumping factor A, and *tst*–toxic shock syndrome toxin.

Out of the 117 identified MDR *S. aureus* strains, 22 strains were selected randomly from each pattern (Table 3) to screen for ARG (*aacA*–*aphD*, *vanA*, *mecA*, *blaZ*, and *apmA*) incidence. The obtained data showed that 63.6% (14/22) of the tested strains harbored the *aacA*-*aphD* gene, 54.5% (12/22) harbored the *mecA* gene, and 50% (11/22) harbored the *blaZ* gene, and confirmed the incidence of MDR in the tested *S. aureus* strains inhabiting retail raw chicken meat samples. However, the *apmA* gene (6/22) and *vanA* gene (5/22) exhibited lower incidence in comparison with the other investigated ARGs (Figure 2). Furthermore, *clfA* (15/22), *tst* (11/22), and *sea* (13/22) exhibited significantly higher incidence in the investigated *S. aureus* strains in comparison with *seb* and *pvl*, which had incidence among the examined *S. aureus* strains with a percentage of 27.3% (6/22) and 13.6% (3/22), respectively.

**Figure 3:**
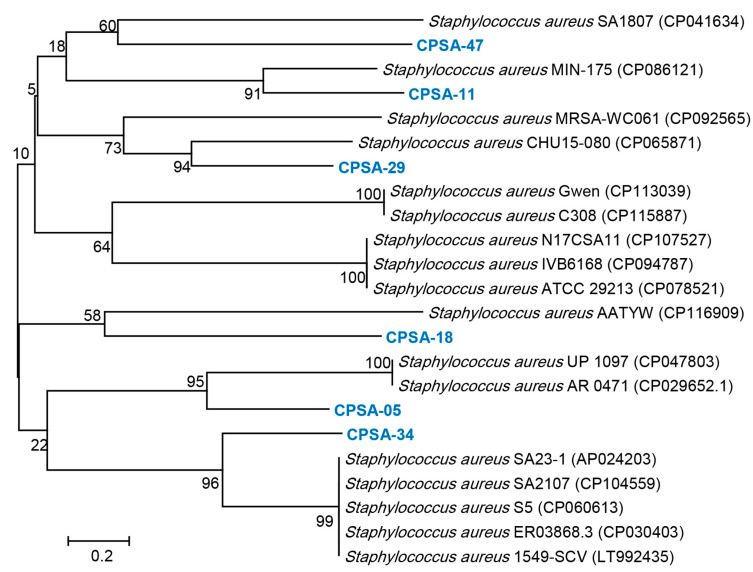
Neighbor-joining tree based on 16S rRNA gene sequences showing the phylogenetic position of the selected resistance and virulence genes producing MDR *S. aureus* strains within closely related taxa. The numbers on tree branches indicate the percentages of bootstrap sampling (≥50%) derived from 1000 replicates. The scale bars indicate substitutions per nucleotide position.

In terms of the molecular identification of *S. aureus*, the phylogenetic tree of the six strains of cluster α (Figure 2) was carried out according to 16S rRNA gene sequencing (Table 4). CPSA-05 showed a 98.7% identity to *Staphylococcus aureus* UP_1097 (CP047803), CPSA-11 showed an identity of 98.15% with *Staphylococcus aureus* Min-175 (CP086121), and CPSA-18 showed a 99.13% identity to *Staphylococcus aureus* AATYW (CP116909). CPSA-29 showed a 98.8% identity with *Staphylococcus aureus* CHU15-080 (CP065871). CPSA-34 and CPSA-47 showed a 98.60 and 9916% identity to *Staphylococcus aureus* 1549-SCV (LT992435) and *Staphylococcus aureus* SA 1807 (CP041634), respectively (Figure 3).

### 2.5. Antimicrobial Activities of Natural Phenolics

MIC values of tested phenolic compounds (thymol, eugenol, carvacrol, protocatechuic acid, and hydroquinone) are listed in Table 5. An important difference in the inhibition of *S. aureus* by the different phenolic compounds was observed in this study. The most effective agent was hydroquinone (mean MIC values of individual strains between 12.5 and 100 μg/mL), followed by carvacrol and thymol. Furthermore, the mean MIC values against *S. aureus* for eugenol and protocatechuic acid were about 6–12 times and 16–24 times higher than hydroquinone (Table 5). The statistical analysis showed that the hydroquinone MICs were significantly lower than thymol, carvacrol, eugenol, and protocatechuic acid at *p* < 0.0001, *p* = 0.0017, *p* = 0.0001, and *p* = 0.0002, respectively.

### 2.6. Effect of Phenolic Compounds on S. aureus Morphology

**Figure 4:**
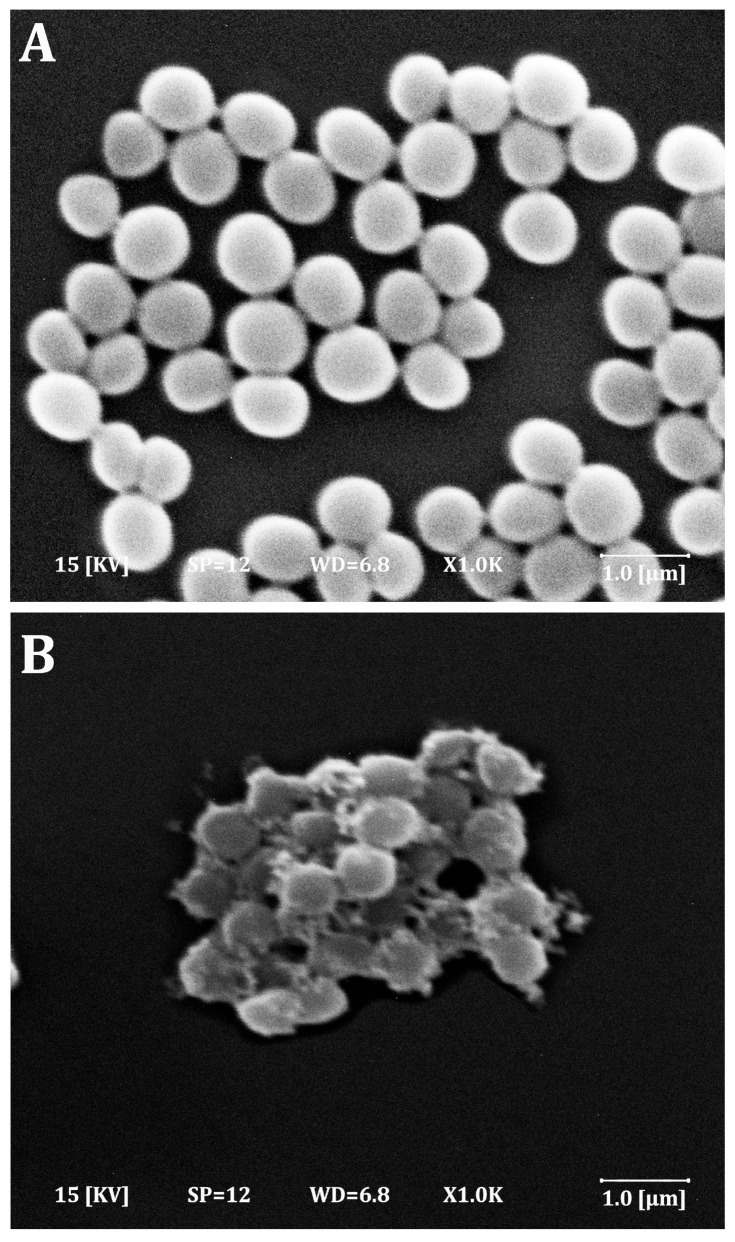
Scanning electron microscope photomicrographs of *S. aureus* before (**A**) and after the treatment with hydroquinone (**B**).

The morphological changes in *S. aureus* cells were detected by SEM analysis. Figure 4 illustrates SEM photomicrographs taken before and after the treatment of *S. aureus* cells with hydroquinone, which exhibited relatively higher antimicrobial activity compared to other tested phenolic compounds. As observed in Figure 4A, bacterial cells without treatment (control) had spherical and regular morphological shapes with smooth surfaces of uniform distribution and size. In contrast, Figure 4B reveals that *S. aureus* cells treated with hydroquinone exhibited wrinkled, irregular outer surfaces, along with adhesion, fragmentation, and aggregation of cellular debris and damaged cells. Moreover, these cells displayed non-uniform sizes and distributions. These results indicate that hydroquinone treatment results in damage to *S. aureus* cells.

## 3. Materials and Methods

### 3.1. Chicken Sample Collection

**Figure 5:**
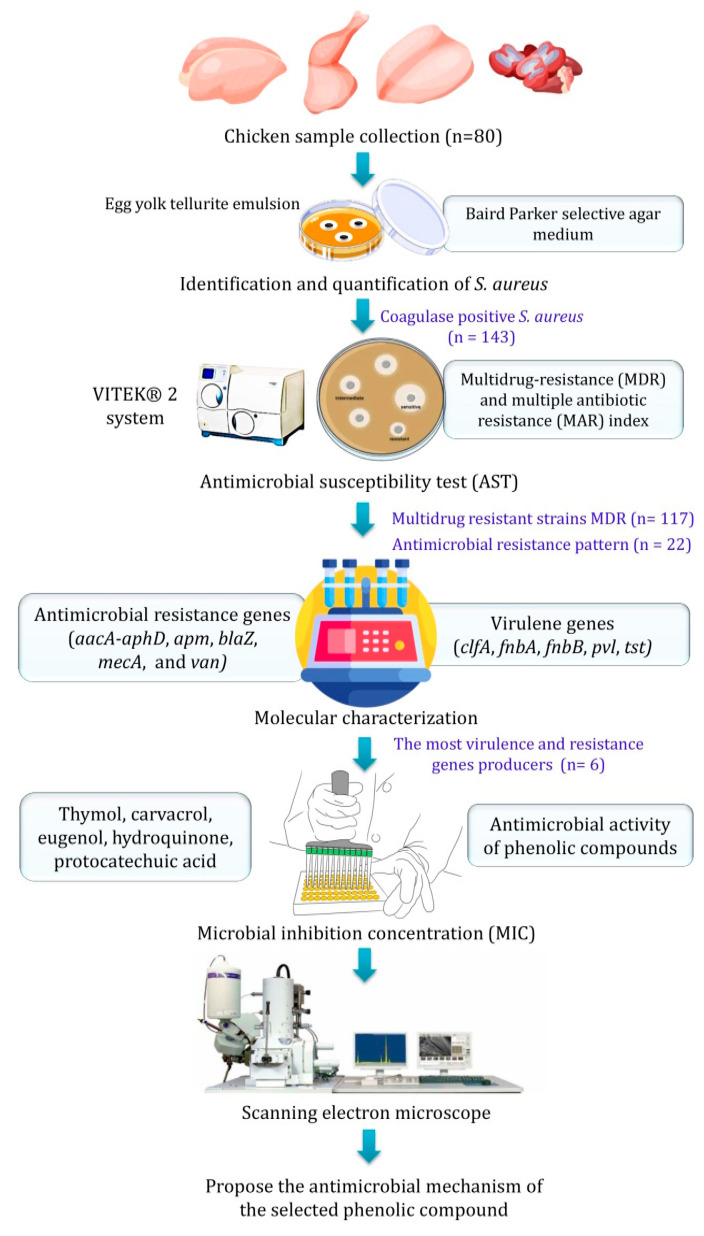
The experimental setup procedures to identify, quantify, and characterize biofilm-producing *S. aureus* from raw chicken meat samples and investigate the activity of various phenolic compounds against the selected *S. aureus* strains.

As illustrated in Figure 5, 80 fresh chicken meat parts, including thighs, breasts, fillets, and giblets purchased from traditional markets in Egypt, were investigated in this study. The purchased samples were transported to the microbiology laboratory in the faculty of veterinary medicine at Zagazig University. To avoid cross-contamination, samples were transported in a sterile plastic bag. Chicken meat samples were aseptically sliced into small pieces for further examination.

### 3.2. Identification and Quantification of S. aureus

The morphological and biochemical identification and quantification of *S. aureus* were carried out following ISO 6888-1:1999/Amd.2:2018 [44] procedures to enumerate CPS with minor modifications (Figure 5). In brief, a weight of 25 g of the purchased raw chicken meat of each sample separately was combined with sterilized buffered peptone water (225 mL; 0.1%, *w*/*v*), and the combination was mixed for 2 min in a stomacher (Masticator IUL, Barcelona, Spain) at 200 rpm. After that, a tenfold serial dilution (10^−5^–10^−8^) was prepared using 1 mL of the homogenate, and a volume of 100 µL of each dilution was spread onto Baird Parker agar medium enriched with egg yolk tellurite emulsion (5%; BP-EY; Oxoid CM1127). After 48 h of incubation at 37 °C, a morphological characterization was conducted to inspect black or dark grey colonies with halo zones and selected as typical colonies for *S. aureus* [45]. Five typical colonies were selected from each BP-EY-positive culture, inoculated into a sterilized brain heart infusion broth (10 mL; Oxoid, UK), incubated at 37 °C for 24 h, and then subjected to hemolytic, coagulase, and catalase activities tests [46]. For further confirmation, it was identified by MALDI TOF MS Biotyper (Brucker, Germany) following the methodological procedure for preparing samples and identification methods of Kmeť et al. [47].

### 3.3. Antimicrobial Susceptibility Test (AST)

An AST was conducted using VITEK^®^ 2 system (bioMérieux, Marcy l’Etoile, Lyon, France) based on the manufacturer’s instructions. In this study, 11 antimicrobial agents (Table 6) were tested against the selected strains. The obtained data were interpreted following CLSI breakpoints [48], as shown in Table 6. MDR is defined as the non-susceptibility to at least one agent in three or more antimicrobial categories [49].

### 3.4. Molecular Identification

The molecular identification of the selected MDR and virulence *S. aureus* strains was conducted by polymerase chain reaction (PCR) specific primer pairs of 16S rRNA F 5′-GTAGGTGGCAAGCGTTATCC-3′ and R 5′-CGCACATCAGCGTCAG-3′ to amplify the 16S rRNA gene of *S. aureus*. PCR programming was begun with an initial denaturation step at 94 °C for 2 min followed by 35 cycles of 94 °C for 30 s, 50 °C for 30 s, and 72 °C for 45 s, ending with a final extension step at 72 °C for 4 min. *S. aureus* ATCC 25,923 was used as a positive control in this experiment. DNA sequencing was analyzed using a BLAST search (http://www.ncbi.nlm.nih.gov/BLAST/ (accessed on 29 April 2023)). A phylogenetic tree was constructed using MEGA software (V. 6) software.

Antimicrobial resistance genes (ARGs) (*blaZ*, *van*, *apmA*, *mecA*, and *aacA*–*aphD*) and various genes involved in virulence (*sea*, *seb*, *pvl*, *clfA*, and *tst*) were investigated by PCR using the listed primers in Table 7. PCR amplification was performed using a Bio-Rad S1000™ Thermal Cycler (Bio-Rad, Fort Worth, TX, USA). For sequencing, purified PCR products were processed at Shanghai Sangon, China. Sequence analysis was performed using Blast search software (http://www.ncbi.nlm.nih.gov/blast (accessed on 29 April 2023)). The evolutionary tree was analyzed and built using MEGA 6 software.

### 3.5. Phenolic Compounds Preparation

Eugenol, thymol, carvacrol, hydroquinone, and protocatechuic acid were all obtained from Sigma–Aldrich, Co. (St. Louis, MO, USA). Stock solutions were freshly prepared following the method described by Gutiérrez-Larraínzar et al. [41]. Briefly, 20 mg of a phenolic compound was dissolved in 1 mL of 5% ethanol and kept away from the light before adding to the Mueller–Hinton broth (MHB; Oxoid Ltd., Hampshire, UK). Briefly, eugenol, hydroquinone, protocatechuic acid, thymol, and carvacrol at a final concentration of 12.8, 12.8, 6.4, 3.2, and 1.6 mg/mL, respectively, and kept away from the light. The compounds were dissolved in 5% ethanol before adding the Mueller–Hinton broth (MHB; Oxoid Ltd.).

### 3.6. Microbial Inhibition Concentration (MIC)

The procedure for performing a microdilution assay to evaluate the MIC values of tested phenolic compounds against pathogenic MDR *S. aureus* strains according to the ISO Standard (20776-1:2006) [57]. After testing the strains’ recovery ability and purity, inoculum for the antimicrobial assay was generated by diluting overnight cultures with sterile MHB to reach 1 × 10^6^ CFU/mL.

The microbial inhibition concentration was examined following the method described by Gutiérrez-Larraínzar et al. [41]. Except for the first row of wells, which were filled with 100 μL of each antimicrobial agent’s stock solution, flat-bottomed 96-well microplates were filled with 50 μL MHB for each well. Different concentrations of antimicrobial compounds were generated in sterile glass tubes from the stock solution, and 50 μL aliquots of each were added into the second and succeeding rows until the last row, which contained just 50 μL of MHB. Each row’s concentration of other antimicrobials was half that of the preceding one. Then, aliquots of standardized inoculum (50 μL) were added to each well to create a 100 μL final volume. In the same plate, a positive control (viable strain) was included. Furthermore, MHB containing 5% ethanol (1/10 *v*/*v*) and a phenolic compound were used as negative controls. The microplates were sealed using a sterile microporous film, mixed manually, and incubated at 37 °C for 24 h. Afterward, the absorbance (OD_620_ nm) was detected using a microplate reader (Bio Kinetics Reader, Bio-Tek Instruments Cultek). MIC was considered the lowest concentration of the tested material that inhibited the visible growth of the tested strain [58]. Three trials on different days were performed for each phenolic compound and strain.

### 3.7. Scanning Electron Microscopy (SEM)

To investigate the morphological changes of *S. aureus* cells before and after treatment, SEM analysis was performed. *S. aureus* was inoculated into Lauria Broth medium (LB) and cultivated at 35 °C for 12 h under shaking conditions. Samples containing *S. aureus* (10^7^ CFU/mL) in LB, along with the selected antimicrobial agent at the MIC value, were incubated at 35 °C for 9 h. Bacterial cells were harvested by centrifugation at 5000 rpm for 10 min at 4 °C. The control was prepared, as previously mentioned, without the antimicrobial agent. Bacterial cells were fixed for 12 h in 2.5% glutaraldehyde. The fixed bacterial cells were washed three times with a 0.1 M phosphate buffer solution (PBS) for 2 h. Then, the bacterial cells were fixed again for 1.5 h with osmic acid and washed three times for 2 h using double-distilled water. Subsequently, the bacterial cells were dehydrated by two rounds of serial dehydration, in 50%, 70%, 80%, 90%, and 100% alcohol solutions, at 15 min intervals, followed by rinsing in isoamyl acetate for 30 min. Finally, the cells were dried by CO_2_ critical point drying (HCP-2, Hitachi, Tokyo, Japan), mounted, and platinized with an ion sputter coater (IB-5) and observed by SEM (JSM-7001F, JEOL, Tokyo, Japan).

### 3.8. Statistical Analysis

The experimental analyses were carried out in triplicate as three independent variables, and the variations observed in the values of each experiment were statistically analyzed using GraphPad Prism version (8.0.2) software. A *t*-test was used to compare the variation between groups.

## 4. Discussion

### 4.1. Phenotypic Characterizations and Incidence of CPS

The results obtained were consistent with numerous reports stating the high prevalence of *S. aureus* in chicken meat in markets [59,60,61]. Wang et al. [45] stated that numerous parameters, including the product storage temperature, isolation process, type of food product, time of sampling, and size, which may cause various contamination by CPS, have been infrequently investigated. However, Narvaez-Bravo et al. [62] found that the difference in *S. aureus* incidence in retail chicken meat might be because of different factors such as slaughtering practices, handling process, geographical location, sampling techniques, and hygienic practices. Consequently, monitoring the incidence of *S. aureus* in chicken meat products under different conditions is an essential practice to ensure product microbiological quality.

### 4.2. Coagulase-Positive Staphylococci Incidence

In this study, all findings suggest the existence of potential sources (environment, material, equipment, method, and workers) of food contamination. These working conditions could promote the proliferation of microorganisms in places of sale and the contamination of chicken. These results conform to those of Guédé et al. [39] who claimed that the diversity of microorganisms present in food products could be because of non-compliance with hygiene instructions, poor sanitary conditions, frequent unhygienic handling, and cross-contamination with materials and packaging. Moreover, similar findings were reported by Abolghait et al. [19] who found that retail chicken meat in the Egyptian market exhibited a high incidence of *S. aureus*. Also, Morshdy et al. [17] stated that out of 60 random samples of chicken meat products, including nuggets, luncheon, and pane, Staphylococci counts were 2.96, 3.14, and 3.32 log CFU/g. Therefore, the authors found that the tested chicken meat products showed unsatisfactory hygienic measures. Hence, strict hygienic procedures should be implemented throughout the processing of chicken meat products to enhance the microbiological quality [17].

### 4.3. Antimicrobial Susceptibility Test

Antibiogram resistance profiles (ARS) were remarkably varied because a total of 22 patterns (P1–P22) were observed among tested strains. P2 was the most common ARP, which was represented by 15 (12.8%) *S. aureus* strains. The strains of the P22 pattern showed a pan drug-resistant phenotype profile (CHL, SAM, CAF, ERY, GEN, IMP, FA, CTX, PMB, TET, and VAN). However, the remaining 21 patterns (P1–P21) were MDR. Moreover, all MDR strains exhibited a high incidence of multiple antibiotic resistance (3–11), and ≥50% of the strains showed multiple resistance to ≥6 antibiotics. Enumerating the MAR index is a useful method for health risk assessment and isolates with values >0.2 suggest it is a possible means of contamination that could be categorized as ‘high risk’ [63]. A MAR index value >0.2 indicates that the isolates originated from an environment where antibiotics were often used [12]. The fluctuated ARPs found in the *S. aureus* strains reveal that *S. aureus* strains may employ numerous mechanisms of resistance simultaneously and may not always follow the same mode of action or behavior to resist various classes of antibiotics.

### 4.4. Molecular Characterization of S. aureus

Various diseases could be successfully treated after developing reliable antimicrobial agents in the 1940s. Still, in recent years, this has become much more challenging because of the organisms’ propensity to develop resistance to the antimicrobials commonly used in medical or industrial applications [64]. In this study, our findings indicate that *S. aureus* strains isolated from chicken meat could be a reservoir of resistance and virulence genes. In this regard, in Nigeria, Igbinosa et al. [65] found that of 368 poultry meat samples, 110 (29.9%) were positive for MRSA. Moreover, Ruzauskas et al. [66] reported that 95% of retail raw chicken meat samples in Lithuanian markets were infected with *Staphylococcus* spp. and observed the incidence of MDR *S. aureus* species. Similar findings were observed in Bangladesh, where 43.5% of *S. aureus* isolates were Methicillin-resistant *S. aureus* [67]. Furthermore, in India, Zehra et al. [68] stated that 52.78% of the isolated strains were MDR and harbored *blaZ*, *mecA*, and *aacA*–*aphD*, tetracycline (*tetK*, *tetL*, and *tetM*), and erythromycin (*ermB* and *ermC*). Additionally, Al-sherees [11] reported that the multiple-aminoglycoside-resistant gene *aacA*/*aphD* provides resistance to aminoglycosides (sisomicin, gentamicin, kanamycin, streptomycin, neomycin, tobramycin, and amikacin). The synthesis of β-lactamases, which are expressed by *blaZ* and can encode the β-lactamase enzyme (penicillinase) which inactivates antibiotics through the hydrolysis of the peptide bond in the *β*-lactam ring, may be the root cause of *S. aureus* resistance to penicillin [69]. Additionally, the *mecA* gene, which encodes a surrogate penicillin-binding protein (PBP2a), may have undergone modification due to penicillin resistance in zoonotic-origin *S. aureus* strains [14,15]. MRSA may pose health risks to consumers of raw chicken meat products. The data in this study showed that 12/22 *S. aureus* strains harbored the *mecA* gene. As a result, sanitary practices should be addressed in Egyptian markets. Liu et al. [70] stated that the *cfr* gene had been previously found in several *Bacillus* spp., *Enterococcus* spp., and *Staphylococcus* spp, which indicates the transferability of this resistance gene. Tsai et al. [71] also reported that the *cfr* gene is a radical SAM (S-adenosyl-L-methionine). Moreover, the *cfr* gene’s mechanism is activated by a binding methyl group to the microbial ribosome, which reduces the binding of several antibiotics to the peptidyl transferase core of the microbial ribosomes. However, Bordeleau et al. [72] found that *apmA* is a unique aminoglycoside antibiotic acetyltransferase encoded gene that produces a 274 amino acid protein that results in resistance to chloramphenicol and/or streptogramin.

While *S. aureus* may generate a wide range of enterotoxins, the exotoxin-encoding genes (*sea* and *seb*) are thought to be responsible for 95% of cases of food poisoning [73]. It is known that SEs are superantigen pyrogenic exotoxin proteins that activate T cells and cause the secretion of large quantities of inflammatory cytokines [74,75]. Also, Jhelum et al. [76] stated that Panton–Valentine leukocidin (PVL)-)-producing *S. aureus* usually causes recurrent skin and soft tissue infections. PVL binds to and kills human neutrophils, causing neutrophil extracellular traps to develop. However, the pathomechanism has yet to be well investigated. In addition to being heat-resistant water-soluble proteins, staphylococcal enterotoxins also maintain their proteolytic resistance properties after ingestion [77]. Staphylococcal fibrinogen-binding protein clumping factor A (*clfA*) is required for endocarditis and arthritis and is a reason for *S. aureus* cells’ development in the bloodstream (platelets and plasma) [78]. TSST-1 is released into the blood, caused by the classical toxin gene *tst*, and results in a variety of severe clinical disorders, including Kawasaki syndrome [79]. Pérez et al. [80] reported that TSST-1 and exotoxin-encoding gene co-production by *S. aureus* might be a factor in developing a more severe immune response syndrome. The emergence and spread of antibiotic resistance are still being tested as options for zoonotic infections by pathogenic *S. aureus* strains.

### 4.5. Antimicrobial Activities of Natural Phenolics

The present study revealed a significant variation in the ability of different phenolic compounds to inhibit *S. aureus*. Hydroquinone emerged as the most potent agent compared with other tested compounds, i.e., carvacrol and thymol demonstrated comparatively lower effectiveness in their inhibitory activity against *S. aureus*. In this regard, Rúa et al. [81] reported that phenolic compounds used as food antioxidants in the European Union are also excellent antibacterial agents combating *S. aureus*, outperforming butylated hydroxyanisole in all aspects. This dual capability might lower the total quantity of meal additives, resulting in more natural products. Gallic acid and hydroquinone, two other phenolic compounds employed as aromatizants in the European Union, are likewise efficient antimicrobials against *S. aureus* and have antioxidant activity. Similar findings were reported by Gutiérrez-Larraínzar et al. [41] who stated that the effect of phenolic compounds varied according to the species and their behavior. The fluctuation in the results could be due to the difference in their genetics and metabolic activities, which affect their response to antibacterial agents. Gutiérrez-Larraínzar et al. [41] reported the maximum concentration level of phenolics in foods (2000 μg/mL) based on the European flavoring industry. Moreover, the authors found that gallic acid is an effective antimicrobial agent with antioxidant activity. Hence, in this study, hydroquinone successfully controlled *S. aureus* (mean MIC value was 54 μg/mL).

**Figure 6:**
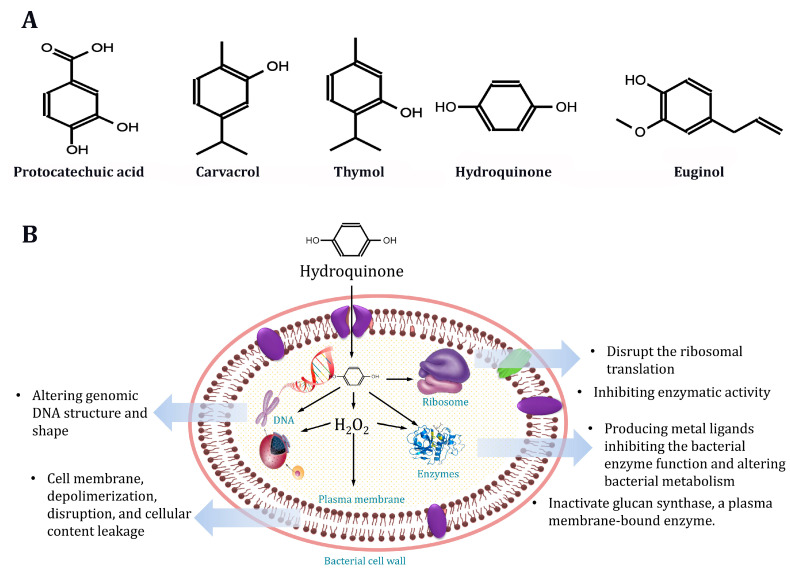
Chemical structures of the phenolic compounds evaluated in this study (**A**) and the proposed antimicrobial activity of hydroquinone against MDR bacteria (**B**).

Generally, hydroquinone showed potential antibacterial activity compared with other tested natural antimicrobial agents (i.e., phenolic compounds) against pathogenic MDR *S. aureus* strains. Hydroquinone had a high MIC value (compared with conventional antimicrobial agents) for some strains, with an average of 54 μg/mL, and showed an antibacterial effect against all tested strains, potentially inhibiting the growth of all pathogenic MDR strains. This effectiveness could be because of the different mechanisms of hydroquinone, which are related to its chemical structure (Figure 6A) and ability to degrade the bacterial cell wall, compared with traditional antibiotics. This might be because of the ability of the bacterial cell to adapt and alter the mechanism of these antibiotics in contrast to hydroquinone [31,69,70]. The variation between the MIC data in this study and those stated in other studies for the phenolic compounds studied might be because of the variations in the applied methodology and the investigated strains’ number and species. These factors are important for attaining real data on antimicrobial agent concentration for the inhibition of pathogenic MDR *S. aureus* strains. Ma et al. [82] recently reported that hydroquinone has a potent antibacterial effect against *S. aureus*, MRSA, and the extended-spectrum β-lactamase *S. aureus* (ESβL-SA).

The antibacterial mechanism demonstrated that hydroquinone might degrade the bacterial cell wall and membrane, disrupt protein synthesis, cause intracellular substance leakage, enhance permeability, change gene expression, and inhibit enzymatic activity, as shown in Figure 6B. Hence, this may be the main reason for the activity of hydroquinone against virulent and MDR *S. aureus* strains isolated from retail raw chicken samples in this study. Although bacterial cells possess a sophisticated multilayered structure that protects them from external stimuli, phenolics may directly attach to and adversely impact their cell membranes. The intricacy of the cell membrane helps bacteria not only survive but also transport nutrients and waste. Gram-positive bacteria are the most susceptible to phenolic chemicals due to the presence of peptidoglycans on their surface and the lack of an outer membrane [83]. In line with these observations, similar results were obtained in the present work, where the tested phenolics showed higher antimicrobial activity against *S. aureus* (Gram-positive) compared to *E. coli* (Gram-negative) strains.

At this level, various reactions occur due to the presence of functional groups in the cell membrane. For example, phenolics can negatively impact the bacterial cell wall by interacting with the hydroxyl groups (-OH) that interact with the peptidoglycans in the cell membrane. The phenolic compound type and the bacterium’s type determine the bacterial resistance to this sort of antibacterial action. Unlike Gram-positive bacteria, Gram-negative bacteria have three cell membrane layers, all of which are resistant to the antibacterial action of phenolic compounds. The high number of phospholipids on the lipophilic outer membrane is linked to this resistance [84]. In this example, the antibacterial mode of action and mechanism include the buildup of hydroxyl groups in lipid bilayers, which disrupts the lipoprotein association and increases cell membrane permeability. Hydroquinone has the potential to compromise membrane integrity, alter cell shape, disrupt cell metabolism, and cause cellular content leakage. Cell death is caused by phospholipid bilayer destruction due to changes in cell division and physiological processes [85]. These findings align with the results obtained in this study, as shown in an SEM micrograph (Figure 5) where the deterioration of the bacterial cell membrane and subsequent cell death were evident. Similarly, Li et al. [86] performed a morphological analysis for treated and untreated *S. aureus* cells using ɛ-poly-lysine and found that the control *S. aureus* cell appeared equal in size and distribution, smooth, and rounded. However, *S. aureus* cells treated with antimicrobial agents exhibited wrinkled and irregular surfaces, accompanied by the aggregation of dead bacterial cells in SEM analysis. These morphological changes can be attributed to the bioactivity of hydroquinone on the bacterial cell wall and membrane, which leads to an increase in the cellular permeability and leakage of the cytoplasmic contents. Moreover, hydroquinone can alter protein synthesis and influence the expression of genes, causing a lethal effect on bacterial cells [82].

Hydroquinone also affects the production and control of DNA in bacteria. Its structure comprises an aromatic ring and hydroxyl groups which enable hydroquinone to interact with the carboxylic or amino groups found in proteins [34]. Because of its effect on cell membrane permeability, hydroquinone can cause the leakage of cellular contents, including DNA. Furthermore, hydroquinone may also attach to genomic DNA, altering its secondary structure and shape [87]. Gogoi et al. [88] and Liu et al. [31] have suggested that the incidence of several hydroxyl groups (OH) might be the main reason for their bioactivity and their microbial toxicological effect, and this improvement in hydroxylation enhances the toxicity effect. In our investigations, hydroquinone, with its two OH groups, demonstrated a superior inhibition of *S. aureus* compared to other examined phenolic substances containing two and one OH group. With respect to protocatechuic acid, the obtained data show that its antibacterial effectiveness is inversely related to its structure (number of OH groups). Notably, the impact of the OH groups may be less important for *S. aureus*, suggesting that this compound could employ a multitargeted action mechanism. However, Hirakawa and Sano [89] proposed that hydroquinone fast autooxidation can generate hydrogen peroxide (H_2_O_2_), which induces hydroquinone antimicrobial activity, possibly through concentration-dependent hydrogen peroxide synthesis. Hydroquinone lacks the ability to directly alter the integrity of bacterial DNA on its own. The H_2_O_2_ synthesis process can alter the bacterial proteins’ expression, resulting in DNA impairment and aberrant transcription [83]. This observation may explain the successful activity of hydroquinone over other tested phenolic compounds.

Additionally, Zhang et al. [34] reported that phenolic chemicals act as antibacterial agents by inhibiting enzymatic activity. Protein–phenolic interactions regulate such expression through a covalent or non-covalent reaction, dependent on protein features (e.g., molecular weight, configuration, hydrophobicity, and amino acid composition). Phenolics may also combine metal ions, producing iron, copper, and zinc ligands that influence bacterial enzyme function and alter bacterial metabolism by inhibiting oxidoreductase, hydrolase, lyase, and transfer enzymes [90]. Phenolic oxidase catalyzes the oxidation of phenolic compounds, resulting in oxidized molecules that inhibit and disrupt glucan synthase (a plasma membrane-bound enzyme). Through covalent changes, oxidized phenolic chemicals permanently alter the structure of glucan synthase [91]. Another route for enzyme inhibition involves the non-specific interactions between phenolic chemicals and protein SH-groups. Because the inhibition efficiency is proportional to the amount of hydroxyl groups, highly oxidized phenolics are more toxic to microorganisms [33]. Moreover, the enzyme inhibition mechanism can occur in conjunction with cytoplasmatic membrane dysfunction and damage. Enzyme systems are found in the membranes of microorganisms, and alterations in the membrane’s lipids can significantly impact enzyme activity [92].

These diverse mechanisms by which phenolic compounds, such as hydroquinone, combat bacterial pathogens may indicate the ability of phenolic compounds and give these compounds potential advantages over conventional antimicrobial agents. The MAR index (Table 3) indicated the inability of conventional antimicrobial agents to inhibit the tested *S. aureus* strains in contrast to the tested phenolic compounds, in particular, hydroquinone, which can affect various microbial organelles. Consequently, this study strongly recommends performing further investigations and experimental studies to comprehensively understand the mechanisms underlying the potential use of phenolic compounds as alternatives to conventional antimicrobial agents.

## 5. Conclusions

The obtained data in this study revealed a high *S. aureus* incidence in raw retail chicken meat in the Egyptian market which can cause FBDs. These pathogens may contribute to common FBDs in Egypt. The antibiotic resistance and pathogenicity were assessed and exhibited severe problems for food industrial applications and quality control. The application of natural compounds as antimicrobial therapy is an expanding field of study in reference to developing a promising technology to combat food containing MDR pathogens. Hydroquinone showed outstanding activity against zoonotic-resistant and virulent *S. aureus* strains. Our findings provide scientific proof of hydroquinone implementation in food safety applications. Hence, hydroquinone applications as a food additive can also serve the industrial application field with particular reference to the inhibition of zoonotic and pathogenic MDR *S. aureus* strains. Therefore, this study suggests initiatives to enhance sanitary standards in Egyptian markets, particularly in traditional markets with higher contamination rates.

## Figures and Tables

**Table 1:** Microbiological quality of the chicken meat parts based on coagulase-positive staphylococci counts.

Tested Samples (No.)	Positive Sample No. (%)	Coagulase-Positive *S. aureus* Counts (log CFU/g)			Microbiological Quality of Tested Samples (log CFU/g)		
		Min.	Max.	Mean ± SE	Satisfactory (<2 log CFU/g) *	Unsatisfactory (2–<4 log CFU/g)	Unacceptable (≥4 log CFU/g)
Breast (20)	20 (100)	0.9	4.8	2.9 ± 0.9	3 (15.0%)	14 (70.0%)	3 (15.0%)
Thigh (20)	19 (95)	1.55	4.8	3.3 ± 0.8	1 (5.3%)	13 (68.4%)	5 (26.3%)
Giblets (20)	17 (85)	0.48	4.12	2.4 ± 1.2	6 (35.3%)	9 (52.9%)	2 (11.8%)
Fillet (20)	17 (85)	2.8	4.4	3.6 ± 0.5	0 (0.0%)	10 (58.8%)	7 (41.2%)
Total (80)	73 (91.3)	0.48	4.8	3.1 ± 0.9	10 (13.7%)	46 (63.0%)	17 (23.3%)

* Egyptian Organization for Specification and Quality Control [42]; Health Protection Agency [43].

**Table 2:** The susceptibility of 143 *S. aureus* strains to the tested antimicrobial agents.

Antimicrobial Agent	Resistant		Intermediate		Susceptible	
	No.	(%)	No.	(%)	No.	(%)
Chloramphenicol (CHL)	72	50.3	2	1.4	69	48.3
Ampicillin/sulbactam (SAM)	73	51.0	2	1.4	68	47.6
Chloramphenicol (CAF)	73	51.0	1	0.7	69	48.3
Erythromycin (ERY)	84	58.7	3	2.1	56	39.2
Gentamicin (GEN)	86	60.1	2	1.4	55	38.5
Imipenem (IMP)	95	66.4	1	0.7	47	32.9
Fusidic acid (FA)	61	42.7	2	1.4	80	55.9
Cefotaxime (CTX)	101	70.6	3	2.1	39	27.3
Polymyxin B (PMB)	94	65.7	1	0.7	48	33.6
Tetracycline (TET)	45	31.5	1	0.7	97	67.8
Vancomycin (VAN)	49	34.3	4	2.8	90	62.9

**Table 3:** Antibiogram resistance profiles (ARPs) of isolated *S. aureus* strains.

Code	Antimicrobial Resistance Pattern	No of Strains	MAR Index
P1	IMP, CTX, and PMB	4	0.27
P2	CHL, CAF, CTX, and PMB	15	0.36
P3	Y and SAM	10	0.36
P4	Y, SAM, and VAN	3	0.45
P5	Y, SAM, and PMB	3	0.45
P6	CAF, ERY, IMP, CTX, and TET	9	0.45
P7	Y, SAM, PMB, and VAN	3	0.55
P8	X, CTX, TET, and VAN	7	0.55
P9	Y, SAM, CTX, and PMB	2	0.55
P10	Y, Z, and CHL	12	0.64
P11	Y, Z, CHL, and VAN	3	0.73
P12	X, Z, GEN, and IMP	7	0.73
P13	Y, Z, SAM, TET, and VAN	4	0.82
P14	Y, Z, SAM, CAF, and TET	6	0.82
P15	Y, Z, CHL, CAF, TET, and VAN	4	0.91
P16	X, Y, Z, and TET	4	0.91
P17	X, Y, Z, and VAN	2	0.91
P18	X, Y, FA, CTX, TET, and VAN	4	0.91
P19	X, Y, FA, PMB, TET, and VAN	3	0.91
P20	X, Z, ERY, IMP, TET, and VAN	5	0.91
P21	X, Z, ERY, IMP, TET, and VAN	2	0.91
P22	X, Y, Z, TET, and VAN	5	1.00

X, CHL, SAM, CAF, Y; ERY, GEN, IMP, Z; FA, CTX, and PMB.

**Table 4:** Molecular identification of the selected pathogenic MDR *S. aureus* based on BLAST comparison to the GeneBank database.

Strain Code	Closest Related Strain	Accession Number	Similarity
CPSA-05	*Staphylococcus aureus* UP_1097	CP047803	98.70
CPSA-11	*Staphylococcus aureus* Min-175	CP086121	98.15
CPSA-18	*Staphylococcus aureus* AATYW	CP116909	99.13
CPSA-29	*Staphylococcus aureus* CHU15-080	CP065871	98.80
CPSA-34	*Staphylococcus aureus* 1549-SCV	LT992435	98.60
CPSA-47	*Staphylococcus aureus* SA 1807	CP041634	99.16

**Table 5:** Minimum inhibitory concentrations (μg/mL) of phenolic compounds against the selected *S. aureus* strains.

Strain Code	Thymol	Carvacrol	Eugenol	Hydroquinone	Protocatechuic Acid
CPSA-5	600 ± 100	320 ± 0	1600 ± 400	100 ± 0	1200 ± 200
CPSA-11	500 ± 100	400 ± 0	2400 ± 400	100 ± 0	800 ± 0
CPSA-18	400 ± 0	600 ± 100	2400 ± 400	12.5 ± 0	1000 ± 300
CPSA-29	600 ± 100	300 ± 0	1600 ± 0	50 ± 0	1200 ± 200
CPSA-34	400 ± 100	300 ± 0	1600 ± 400	12.5 ± 0	600 ± 100
CPSA-47	400 ± 100	400 ± 100	2400 ± 400	50 ± 0	800 ± 0

Values are the mean of at least two experiments in triplicate ± error standard deviation of the mean.

**Table 6:** Antimicrobial agents and interpretive zone chart (CLSI, 2017) [48].

Antimicrobial Group	Antimicrobial Agent	Concentration (µg/mL)	Abb.	Breakpoints		
				S	I	R
Phenicol	Chloramphenicol	30	CHL	≥18	13–17	≤12
*β*-lactam	Ampicillin/sulbactam	10/10	SAM	≥2	-	≤2
Chloramphenicol	Chloramphenicol	30	CAF	≥18	13–17	≤12
Macrolides	Erythromycin	15	ERY	≥23	14–22	≤13
Aminoglycosides	Gentamicin	10	GEN	≥15	13–14	≤12
Carbapenem	Imipenem	10	IMP	≥19	16–18	≤15
Fusidane	Fusidic acid	10	FA	≥22	20–21	≤19
Cephalosporins	Cefotaxime	30	CTX	≥26	-	≤2
Polymixins	Polymyxin B	300	PMB	≥12	9–11	≤8
Tetracycline	Tetracycline	30	TET	≥15	12–14	≤11
Glycopeptide	Vancomycin	30	VAN	≥17	15–16	≤14

**Table 7:** Primers were used in the molecular characterization of virulence and resistance genes of *S. aureus* in this study.

Target Gene	Sequence (5′-3′)	References
*sea*	F-GGTTATCAATGTGCGGGTGG	[50]
	R-CGGCACTTTTTTCTCTTCGG	
*seb*	F-GTATGGTGGTGTAACTGAGC	[50]
	R-CCAAATAGTGACGAGTTAGG	
*clfa*	F-ATTGGCGTGGCTTCAGTGCT	[51]
	R-CGTTTCTTCCGTAGTTGCATTTG	
*tst*	F-TTCACTATTTGTAAAAGTGTCAGACCCACT	[52]
	R-TACTAATGAATTTTTTTATCGTAAGCCCTT	
*pvl*	F-ATCATTAGGTAAAATGTCTGGACATGATCCA	[52]
	R-GCATCAASTGTATTGGATAGCAAAAGC	
*aacA*–*aphD*	F-TAATCC AAG AGC AAT AAG GGC	[53]
	R-GCCACACTATCATAACCACTA	
*vanA*	F-GGCAAGTCAGGTGAAGATG	[54]
	R-ATCAAGCGGTCAATCAGTTC	
*mecA*	F-AGAAGATGGTATGTGGAAGTTAG	[54]
	R-ATGTATGTGCGATTGTATTGC	
*blaZ*	F-ACTTCAACACCTGCTGCTTTC	[55]
	R-TGACCACTTTTATCAGCAACC	
*apmA*	F-CGTTTGCTTCGTGCATTAAA	[56]
	R-TTGACACGAAGGAGGGTTTC	

## Data Availability

All obtained data in this work are included in the submitted manuscript.

## Supplementary Materials

The following supporting information can be downloaded at: https://www.mdpi.com/article/10.3390/molecules28186742/s1, Figure S1: The susceptibility patterns (in percentages) against the tested antibiotics against *S. aureus* strains isolated from raw chicken meat samples.
