# Supplementary material for: Potential of Natural Phenolic Compounds as Antimicrobial Agents against Multidrug-Resistant Staphylococcus aureus in Chicken Meat
Source: Molecules. 2023 Sep 21;28(18):6742. doi: 10.3390/molecules28186742 (PMC10535414; doi:10.3390/molecules28186742)
Supplement: Supplementary file 1 [file molecules-28-06742-s001.zip › molecules-2575934-supplementary.pdf]

Supplementary data

Potential of Natural Phenolic Compounds as Antimicrobial Agents against Multidrug-Resistant *Staphylococcus aureus* in Chicken Meat

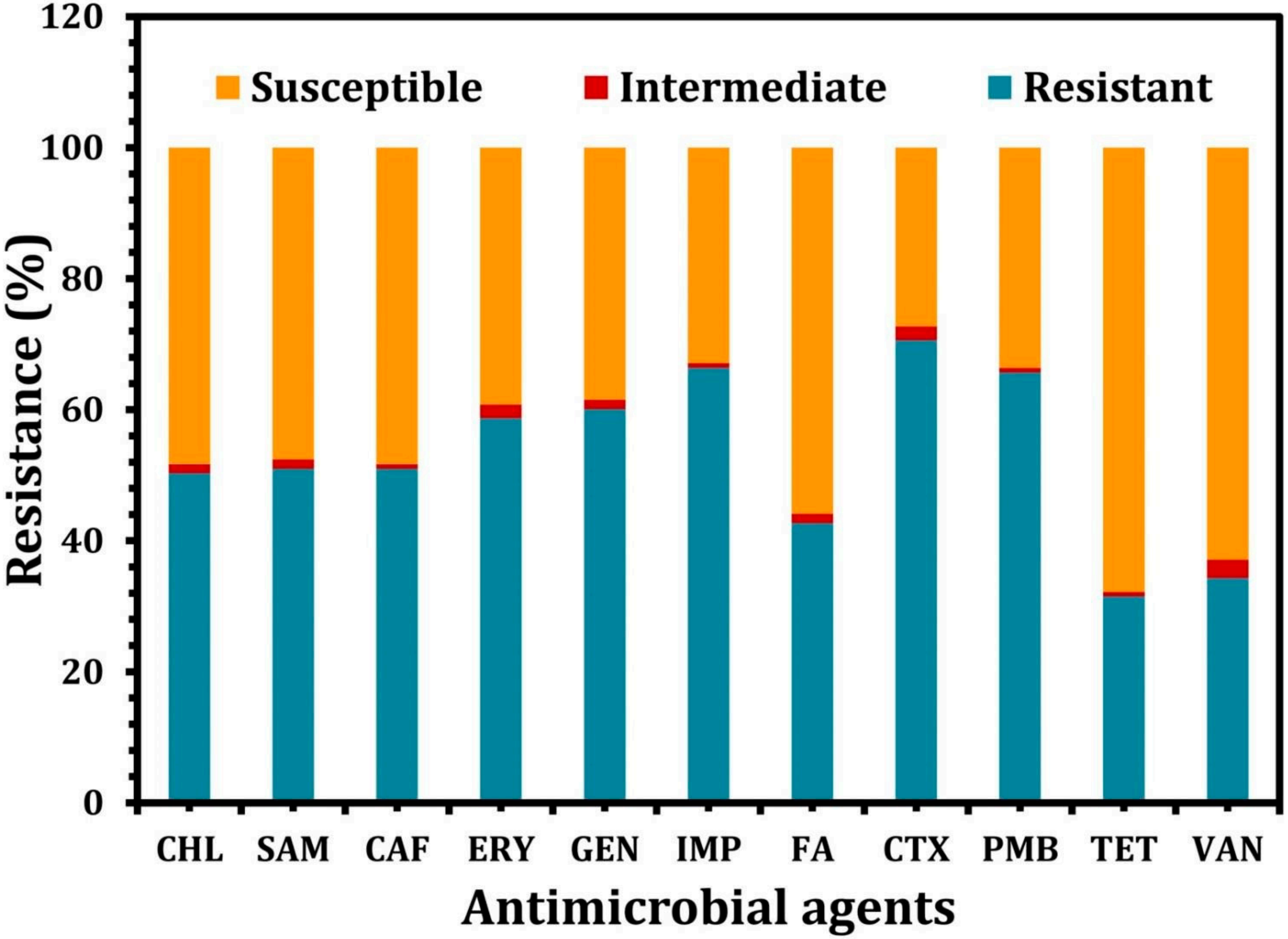

Figure S1. The susceptibility patterns (in percentages) against the tested antibiotics against *S. aureus* strains isolated from raw chicken meat samples.
